# Antibiotic export by efflux pumps affects growth of neighboring bacteria

**DOI:** 10.1038/s41598-018-33275-4

**Published:** 2018-10-11

**Authors:** Xi Wen, Ariel M. Langevin, Mary J. Dunlop

**Affiliations:** 1 Biomedical Engineering Department, Boston University, Boston, MA 02215 USA; 2 Biological Design Center, Boston University, Boston, MA 02215 USA

## Abstract

Cell-cell interactions play an important role in bacterial antibiotic resistance. Here, we asked whether neighbor proximity is sufficient to generate single-cell variation in antibiotic resistance due to local differences in antibiotic concentrations. To test this, we focused on multidrug efflux pumps because recent studies have revealed that expression of pumps is heterogeneous across populations. Efflux pumps can export antibiotics, leading to elevated resistance relative to cells with low or no pump expression. In this study, we co-cultured cells with and without AcrAB-TolC pump expression and used single-cell time-lapse microscopy to quantify growth rate as a function of a cell’s neighbors. In inhibitory concentrations of chloramphenicol, we found that cells lacking functional efflux pumps (Δ*acrB*) grow more slowly when they are surrounded by cells with AcrAB-TolC pumps than when surrounded by Δ*acrB* cells. To help explain our experimental results, we developed an agent-based mathematical model, which demonstrates the impact of neighbors based on efflux efficiency. Our findings hold true for co-cultures of *Escherichia coli* with and without pump expression and also in co-cultures of *E*. *coli* and *Salmonella typhumirium*. These results show how drug export and local microenvironments play a key role in defining single-cell level antibiotic resistance.

## Introduction

Despite intensive study, antibiotic resistance remains an essential problem, in part due to the myriad of mechanisms by which cells can evade drug treatment. Classical tests, such as measurements of the minimum inhibitory concentration (MIC), are important for quantifying drug resistance, but can obscure single-cell level differences in resistance^1^. This is a significant problem because cell-to-cell differences in antibiotic resistance can establish concentration gradients, which can accelerate the resistance acquisition process^2,3^. In addition, subpopulations of antibiotic resistant or tolerant cells can decrease treatment efficacy^4,5^.

Individual cells can exhibit phenotypic differences in drug resistance even in the absence of community-level effects. For example, persister cells use dormancy or slow growth to evade antibiotic treatment^1^. Single-cell level resistance can also affect group growth. For instance, *Streptococcus pneumoniae* cells with chloramphenicol acetyltransferase can deactivate chloramphenicol, resulting in a decrease in both the intracellular and environmental chloramphenicol concentrations^6^. Bacteria also transiently express resistance-conferring genes such as drug export pumps or those that modify membrane permeability, resulting in cell-to-cell difference in susceptibility^4,7^.

Antibiotic efficacy can also be dependent on community-level phenomena. For example, the inoculum effect describes the cell density dependence of the MIC, where more dense cultures are less susceptible to antibiotics resulting in increases in the MIC^8,9^. Cell density plays an essential role in influencing group behaviors, such as quorum sensing and biofilm formation, which in turn can dramatically increase the antibiotic resistance of the population^10,11^. Furthermore, certain cells within a community may exhibit altruistic behavior, such as those that release resistance proteins upon death to enable other cells to survive^10,12^. These examples highlight the importance of cellular interactions and collective behavior in antibiotic resistance.

Bacterial efflux pumps are an important source of multidrug resistance^13,14^. These pumps export antibiotics from the cell, increasing their antibiotic resistance. Their expression can be taxing, reducing growth and imposing a fitness cost^15,16^; therefore, their expression is often regulated to limit the burden. The primary multidrug resistance efflux pump in *E*. *coli* is AcrAB-TolC. This pump is composed of three proteins that span the inner and outer cell membrane: a periplasmic linker protein AcrA, the inner membrane efflux transporter AcrB, and the outer membrane channel TolC^17^. Knocking out *acrB*, the pump protein responsible for substrate recognition and export via the proton motive force, leads to a significant increase in antibiotic susceptibility^14,18^. For instance, the MIC of *E*. *coli* Δ*acrB* to chloramphenicol is an eighth of that of wild type cells^19^. Complementing Δ*acrB* with the *acrAB* operon is sufficient to restore drug resistance^15^. Efflux pumps have been recognized to play a major role in clinical isolates in the emergence of resistant strains of *E*. *coli*, *Salmonella enterica*, and other pathogens, and thus have been identified as clinical targets^20,21^.

Recent studies have shown that AcrAB-TolC expression is heterogeneous across populations^22,23^, suggesting that differential pump expression exists even within isogeneic populations. Since the cost and benefit of expressing pumps can both be significant, these cell-to-cell differences may have important implications for bacterial populations. Here, we asked how efflux pump export of antibiotics affects the growth of neighboring cells and, ultimately, the composition of the population.

To accomplish this, we focused on differential expression of *acrAB*. We monitored single-cell growth rates using time-lapse microscopy, and analyzed growth of cells as a function of whether their neighbors have AcrAB-TolC efflux pumps. We found that individual bacteria that are surrounded by AcrAB-expressing neighbor cells will tend to grow more slowly than when the same cells are surrounded by Δ*acrB* neighbors under antibiotic exposure. By developing a mathematical model, we were able to characterize this effect and predict the cell growth in the presence of a different antibiotic. Furthermore, we tested co-cultures of *E*. *coli* and *S*. *enterica* serovar Typhimurium (hereafter referred to as *S*. *typhimurium*) and observed the same neighbor dependence, which has implications for the broader relevance of our findings since these results likely extend to mixed-species communities. This work contributes additional evidence for the critical role of single-cell level effects in antibiotic resistance.

## Results

**Figure 1:**
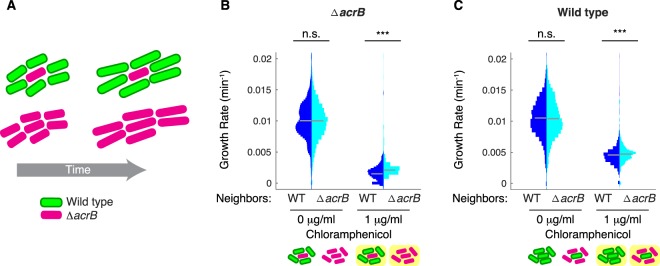
Neighbors with pumps impact cell growth. (**A**) Schematic showing when Δ*acrB* cells are surrounded by cells with AcrAB-TolC pumps they grow more slowly than when surrounded by other Δ*acrB* cells. (**B**) Growth rates of wild type cells expressing *gfp* (WT-GFP) and Δ*acrB* cells expressing *rfp* (Δ*acrB-*RFP). Cells were mixed in ratios of 5:1 and 1:5 and the growth rate of Δ*acrB-*RFP cells was then quantified for the two different ratios. (**C**) Growth rates of wild type cells, given WT-GFP or Δ*acrB-*RFP neighbors. For (**B**,**C**) statistical significance was calculated using the Kolmogorov-Smirnov test, where ***p < 0.001, n.s.: not significant. Gray bars show mean growth rate. Distribution mean, standard deviation, and p-values are listed in Table S1. Plot axis limits were set to show >97% of cells; however full data set including outliers and n values (number of cells) for each are shown in Fig. S2. Schematics under (**B**,**C**) show the type of neighbors surrounding the cell in the middle whose growth rate is calculated. Background color indicates presence of antibiotics.

To examine the effect of drug efflux on neighboring cells, we designed an experiment where Δ*acrB* cells were surrounded either wild type cells containing functional AcrAB-TolC pumps or by identical Δ*acrB* cells (Fig. 1A). We hypothesized that Δ*acrB* cells which had wild type neighbors would experience a higher local concentration of antibiotics due to drug efflux in their immediate vicinity, leading to a reduced growth rate relative to cells with neighbors lacking pumps. To test this, we conducted experiments with *E*. *coli* growing on agarose pads and measured single cell growth rates under different levels of antibiotic exposure.

To visualize the two cell types, we labeled the Δ*acrB* cells with red fluorescent protein (denoted Δ*acrB*-RFP) and wild type cells with green fluorescent protein (WT-GFP). Chloramphenicol is a broad-spectrum antibiotic which diffuses through the bacterial cell membrane and reversibly binds to the ribosome to inhibit protein synthesis. We quantified the growth rates of Δ*acrB*-RFP cells surrounded by either WT-GFP or Δ*acrB*-RFP neighbors. To do this, we mixed Δ*acrB*-RFP with WT-GFP cells in ratios of 1:5 and 5:1 to bias the community structure. Growth rates for cells were similar for both ratios for conditions with no chloramphenicol. However, under chloramphenicol treatment just below the MIC (1 μg/ml, Fig. S1), we found that the growth rate of Δ*acrB* cells with WT-GFP neighbors was lower than those with Δ*acrB*-RFP neighbors (Fig. 1B), indicating that the influence of drug efflux by neighboring cells is important in local growth inhibition. When we compared the growth of WT-GFP cells with WT-GFP or Δ*acrB*-RFP neighbors, we observed more modest differences in growth rates under chloramphenicol treatment. This is likely because cells with pumps are able to maintain low intracellular antibiotic concentrations regardless of their neighbors (Fig. 1C).

**Figure 2:**
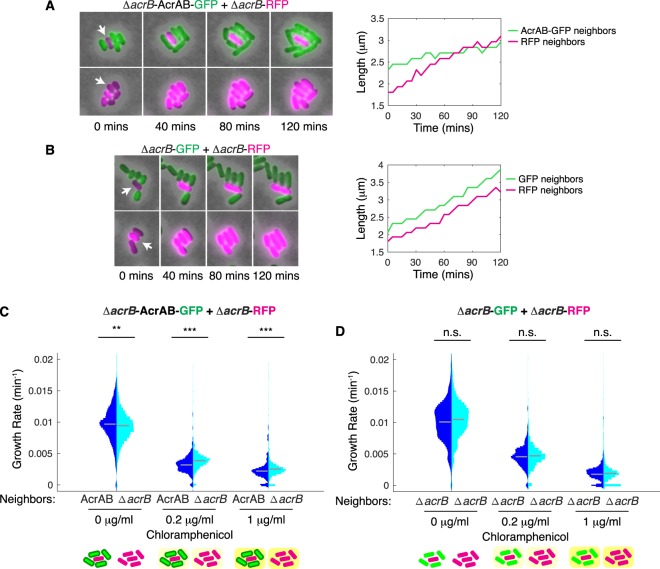
Δ*acrB* cells with and without *acrAB* complementation show neighbor-dependent differences in growth. (**A**) Δ*acrB-*RFP and AcrAB*-*GFP cells were mixed in ratios of 1:5 and 5:1 and grown on agarose pads with 0.2 µg/ml chloramphenicol. Left panel is representative series of time-lapse images showing growth of a Δ*acrB-*RFP cell surrounded by AcrAB*-*GFP neighbors. Right panel shows the cell length over time for the cell indicated with an arrow in the left panel. (**B**) Δ*acrB-*RFP and Δ*acrB-*GFP cells for conditions as described in (**A**). Length data for all cells for conditions from (**A**,**B**) are shown in Fig. S3. **(C)** Growth rates of Δ*acrB-*RFP cells with either AcrAB-GFP or Δ*acrB-*RFP neighbors quantified at different chloramphenicol concentrations. (**D**) Growth rates of Δ*acrB*-RFP cells with either Δ*acrB*-GFP or Δ*acrB*-RFP neighbors. Statistical significance was calculated using the Kolmogorov-Smirnov test. ***p < 0.001; **p < 0.01; n.s.: not significant. Gray bars show mean growth rate. Distribution mean, standard deviation, and p-values are listed in Table S1. Full data set including outliers and n values are shown in Fig. S2. Schematics under (**C**,**D**) show the type of neighbors surrounding the cell in the middle whose growth rate is calculated. Background color indicates antibiotic concentration.

Building upon these results, we next conducted a series of experiments where we used Δ*acrB* as the strain background for both types of cells in the co-culture, allowing us to isolate the effect of efflux pumps independent of endogenous regulation. We tested microbial communities with Δ*acrB*-RFP cells and a Δ*acrB* strain overexpressing *acrAB*, which we labeled with green fluorescent protein (denoted AcrAB-GFP). We then monitored the growth of the Δ*acrB*-RFP cells surrounded by either AcrAB-GFP or Δ*acrB*-RFP neighbors. As before, we found that Δ*acrB*-RFP cells grow more slowly when they are in the vicinity of AcrAB-GFP neighbors than when they are surrounded by Δ*acrB*-RFP neighbors (Fig. 2A). Differences in the growth rate are apparent in measurements of cell length over time. As a negative control, we also measured Δ*acrB*-RFP cells mixed with Δ*acrB*-GFP cells and found no differences in growth rate (Fig. 2B).

To confirm our findings across measurements of hundreds of individual cells, we quantified the growth rates of single cells with Δ*acrB*-RFP or AcrAB-GFP neighbors. We found statistically significant differences in the growth rates in conditions where antibiotics were applied (Fig. 2C). In addition, we observed a shift in the mean growth rate in the opposite direction without antibiotic treatment, indicative of the cost of efflux pump expression. Under sub-MIC levels of chloramphenicol (0.2 μg/ml), the neighbor effect was more apparent than chloramphenicol concentrations near the MIC (1 μg/ml). This is likely because at the higher antibiotic concentration growth of both Δ*acrB*-RFP and AcrAB-GFP cells is impacted by chloramphenicol treatment. As expected, control experiments with Δ*acrB*-RFP and Δ*acrB*-GFP cells showed no statistical difference in growth rates, regardless of the antibiotic concentration (Fig. 2D). These results indicate that the AcrAB-TolC efflux pump plays a role in attenuating growth of neighboring cells in conditions where antibiotics are present.

**Figure 3:**
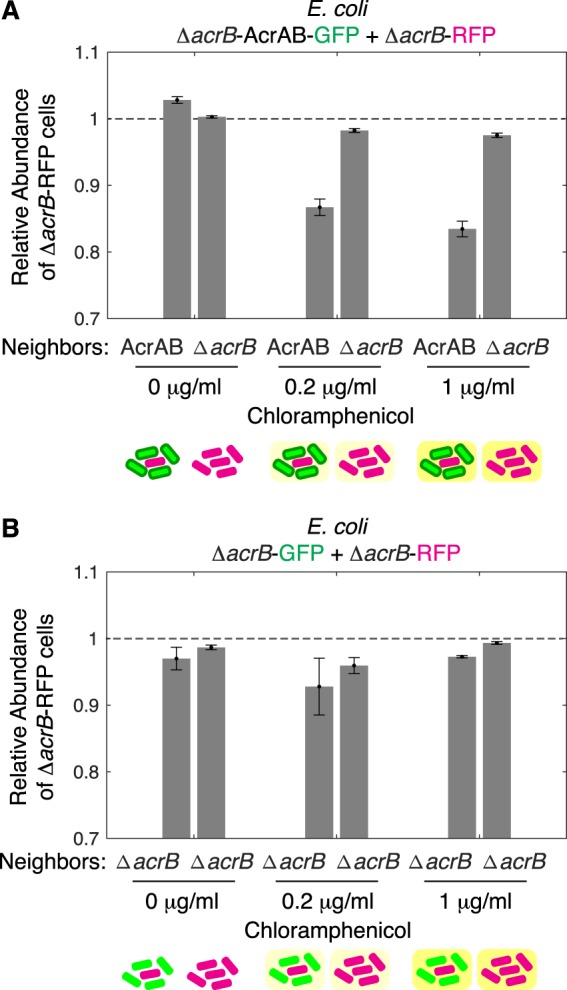
Relative abundance of Δ*acrB* cells decreases when they have AcrAB*-*GFP neighbors. (**A**) Relative abundance was calculated using the data set in Fig. 2C, where we define relative abundance as the fraction of the biomass Δ*acrB*-RFP cells make up at the end, divided by their fraction at the start. (**B**) Relative abundance calculated using the data set in Fig. 2D. Dashed line at one indicates value if there is no change in the abundance of Δ*acrB*-RFP cells over time. Error bars show standard deviation between replicates. Schematics under plots show the type of neighbors surrounding the cell in the middle whose growth rate is calculated. Background color indicates antibiotic concentration.

Since competition will change the composition of cells in mixed species communities, we next extended our analysis to ask what the implications were for co-cultures. To do this, we compared the biomass of the Δ*acrB*-RFP cells at the start of the co-culture experiment to the end. More specifically, we quantified the relative abundance of the Δ*acrB*-RFP cells by comparing what fraction of the biomass they made up at the end divided by the fraction at the start. Thus, if there is no change in the composition of the co-culture then the relative abundance will be one; values below one correspond to AcrAB-GFP cells outcompeting the Δ*acrB*-RFP cells. When no antibiotic was applied we found that Δ*acrB*-RFP and AcrAB-GFP cells grew similarly and the relative abundances of the two strains were maintained near one (Fig. 3A). However, under chloramphenicol treatment the relative abundance of the Δ*acrB*-RFP cells decreased when they were surrounded by AcrAB-GFP cells, but not when they were in close proximity with other Δ*acrB*-RFP cells. We note that under these conditions there are still AcrAB-GFP cells, but since they are mixed in a ratio of 5:1, the AcrAB-GFP cells are comparatively rare. Control experiments with Δ*acrB*-RFP and Δ*acrB*-GFP co-cultures had relative abundance values near one regardless of the chloramphenicol concentration (Fig. 3B). Overall, these results indicate that proximity related inhibition from drug efflux can lead to rapid changes in the community composition.

**Figure 4:**
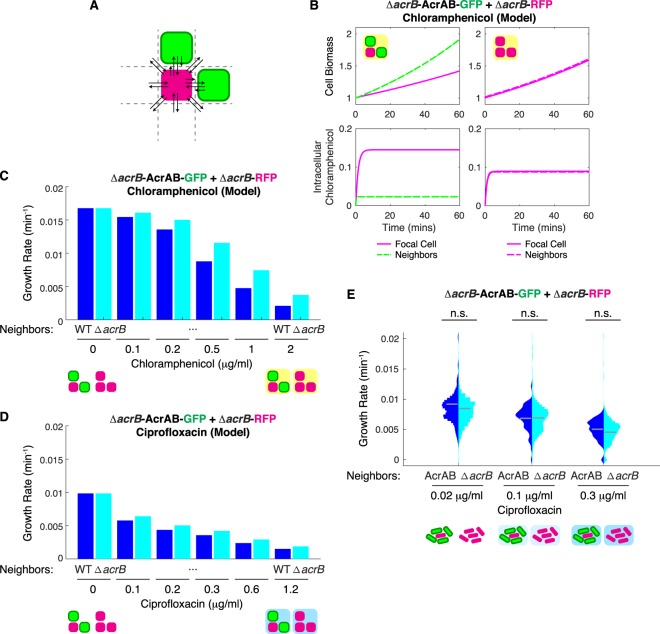
Model predicts cell growth rate differences under antibiotic conditions. (**A**) Schematic depicting the spatial relationship between the focal cell in the center, its neighbors, and the environment. (**B**) Biomass and intracellular chloramphenicol concentration of Δ*acrB* cells with wild type neighbors or Δ*acrB* neighbors simulated in an environment with 0.1 µg/mL of chloramphenicol. **(C**) Cell growth of Δ*acrB* cells with different chloramphenicol concentrations given wild type or Δ*acrB* neighbors. Growth rate is calculated as the average change in biomass divided by the time simulated. Model parameters and initial conditions are listed in Table S2. (**D**) Cell growth under ciprofloxacin treatment for the same cell configurations as in (**C**). **(E)** Δ*acrB-*RFP and AcrAB-GFP cells were mixed in different ratios (1:5 or 5:1) and grown on agarose pads with ciprofloxacin. Statistical significance was calculated using the Kolmogorov-Smirnov test, where n.s.: not significant. Gray bars show mean growth rate. Distribution mean, standard deviation, and p-values are listed in Table S1. Full data set including outliers and n values for each are shown in Fig. S2. Schematics under (**C**–**E**) show the type of neighbors surrounding the cell in the middle whose growth rate is calculated. Background color indicates presences of antibiotics.

To understand the impact of antibiotic export on neighboring cells, we developed a mathematical model to describe cell growth. The agent-based model applies a fixed spatial architecture to describe cell proximity. Within each cell, we used a system of ordinary differential equations to model changes in the intracellular antibiotic concentration due to drug efflux (Fig. 4A). Model parameters were estimated from measurements of cell density in the presence of antibiotics (Fig. S1). We found that cell growth and the intracellular antibiotic concentration are strongly influenced by the type of neighbors in the simulation (Fig. 4B). We next simulated a range of chloramphenicol concentrations and found that the growth rate decreased significantly for cells with higher efflux compared to cells with Δ*acrB* neighbors (Fig. 4C), in good agreement with the experimental results (Fig. 1B).

A key finding of the model is that the efflux rate is proportional to the neighbor effect. In other words, if the AcrAB-TolC pump exports a specific antibiotic well, then the neighbor effect will be more apparent than if the pump does not export it well. To test this, we conducted additional modeling and experiments with ciprofloxacin, which is a substrate of the AcrAB-TolC pump, but has a smaller fold reduction of the MIC than chloramphenicol for Δ*acrB* cells (Fig. S1B). Using parameter fits from experimental data, we lowered the efflux rate of wild type cells to model the lower efflux efficiency for ciprofloxacin. The simulated results show a decrease in the impact of neighbors on the focal cell’s growth rate (Fig. 4D). We confirmed this experimentally with ciprofloxacin, observing modest, but not statistically significant differences between the different neighboring cells (Fig. 4E). In an extension to the model, we explored how the neighborhood affected the focal cell’s growth rate. We observed that the overall number of neighbors was an important determining factor of the focal cell’s growth rate and the exact spatial arrangement of the neighbors played only a minor role (Fig. S4).

**Figure 5:**
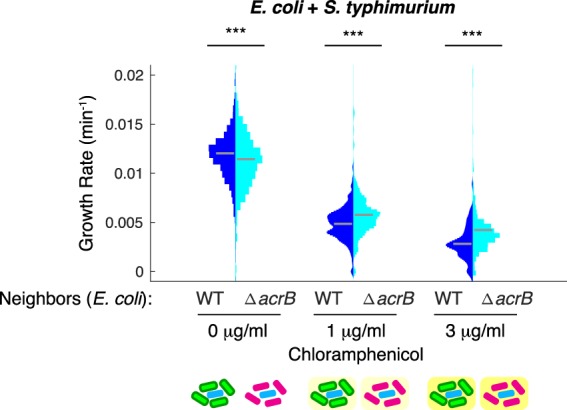
*E*. *coli* and *S*. *typhimurium* co-culture. *S*. *typhimurium* cells were mixed with either WT-GFP or Δ*acrB*-RFP *E*. *coli*. Statistical significance was calculated using the Kolmogorov-Smirnov test. ***p < 0.001. Gray bars show mean growth rate. Distribution mean, standard deviation, and p-values are listed in Table S1. Full data set including outliers and n values for each are shown in Fig. S2. Schematic under plot shows the type of neighbors surrounding the cell in the middle whose growth rate is calculated. Background color indicates antibiotic concentration.

In microbial communities bacterial cross-species interactions are common. Therefore, we tested whether the neighbor effect was limited to our single-species co-cultures with *E*. *coli* or if it extended to cross-species interactions. *E*. *coli* (e.g. ETEC or STEC) and *S*. *typhimurium* are both foodborne pathogens and their co-existence can lead to mixed biofilm formation and a higher resistance against sanitization^24^. We investigated the growth of *S*. *typhimurium* co-cultured with *E*. *coli* WT-GFP or Δ*acrB*-RFP under conditions with and without chloramphenicol. Consistent with our results from the single-species co-cultures, we observed that *S*. *typhimurium* grows more slowly with *E*. *coli* WT-GFP neighbors than *E*. *coli* Δ*acrB*-RFP neighbors (Fig. 5). These results indicate that the neighbor effect generalizes to cross-species interactions.

## Discussion

Single cell level effects are important for bacterial growth and survival under antibiotic treatment. Here we focused on differences in antibiotic efflux as a mechanism for generating cell-to-cell differences in antibiotic survival. This work is motivated by recent studies showing that efflux pump expression is variable across cells within a bacterial population^22,23^. Using detailed quantitative measurements of single cell growth rates, we asked how differences in drug efflux affect the growth of neighboring cells. We found that Δ*acrB* cells have a lower growth rate when surrounded by cells with the AcrAB-TolC pump than when surrounded by like Δ*acrB* cells. This effect leads to a rapid shift in the community composition towards more resistant cells that occurs within a small number of generations. Further, the effect extends to *E*. *coli* and *S*. *typhumirium* co-cultures, suggesting that these findings are likely to be broadly relevant for mixed-species communities and stress tolerance mechanisms that work by exporting antibiotics or other compounds into the immediate vicinity.

Efflux pump expression can be burdensome to cells and there is a tradeoff between the benefit of pumps and their cost^15^. Under the conditions we tested here, the cost of pumps was modest and conditions with no antibiotics produced only minor differences in growth rates between Δ*acrB*-RFP and AcrAB-GFP cells; however, we note that as experiment durations are extended this burden will become more apparent. These cost and benefit tradeoffs will likely depend on the environment, as cells balance the burden of pump expression, the impact of their neighbors, and the local antibiotic concentration to maximize growth.

In the future, it will be interesting to study the interaction between drug efflux and other antibiotic resistance mechanisms that function at the single-cell level. Also, efflux pump expression is stochastic and can change over time in individual cells^22,23^, suggesting the potential for experiments that quantify how these dynamics affects growth of neighboring bacteria. The implications for the eventual evolution of permanent genetic changes that lead to antibiotic resistance are also an interesting area for future research. Single cell level effects and how bacteria interact, including their proximity, can have a profound impact on whether antibiotics are effective.

## Methods

### Strains and plasmids

We used BW25113 as the wild type strain of *E*. *coli*. BW25113 Δ*acrB* was derived from the Keio collection strain JW0451 (BW25113 Δ*acrB*::kan^R^)^25^, and we removed the kanamycin resistance marker using the pCP20 plasmid^26^. For *Salmonella* co-culture experiments we used the model strain *S*. *typhimurium* LT2^27^.

Plasmids were constructed using the Gibson assembly method^28^. To distinguish the strains, we used fluorescent reporters encoded on plasmids. For RFP, we used the plasmid pBbA5k-rfp^29^, for GFP we used pBbA5k-sfgfp^15^, and for AcrAB-GFP we used pBbA5k-acrAB-sfgfp^15^, where *acrAB* and *sfgfp* are transcriptionally fused. All plasmids described above have an IPTG-inducible P_lacUV5_ promoter controlling gene expression, a medium copy p15A origin of replication, and kanamycin resistance marker. The plasmids were transformed into either the *E*. *coli* wild type strain (pBbA5k-sfgfp to make WT-GFP), *E*. *coli* Δ*acrB* strain (pBbA5k-rfp for Δ*acrB*-RFP; pBbA5k-acrAB-sfgfp for AcrAB-GFP; pBbA5k-sfgfp for Δ*acrB*-GFP), or *S*. *typhimurium* strain (pBbA5k-rfp).

### Growth conditions

*E*. *coli* and *S*. *typhimurium* were cultured in Luria Broth (LB) medium. For all experiments, overnight cultures were inoculated from a single colony in LB with 30 μg/ml kanamycin for plasmid maintenance. Overnight cultures were then grown at 37 °C with orbital shaking at 200 rpm. Before experiments, cultures were refreshed 1:50 in LB with kanamycin and grown at 37 °C with orbital shaking. After 5 h, we added 100 μM IPTG and then incubated an additional 2 h to induce fluorescent protein or AcrAB expression. For *S*. *typhimurium*, 100 μM IPTG was added after cultures were refreshed for 0.5 h and cells were grown for an additional 2 h induction. Co-cultures were mixed in ratios of 1:5 and 5:1 each for Δ*acrB*-RFP and WT-GFP or Δ*acrB*-RFP and AcrAB-GFP experiments (and control with Δ*acrB*-RFP and Δ*acrB*-GFP).

### Time-lapse microscopy

For imaging experiments, the co-cultures were placed on an agarose pad with 100 μM IPTG and with either 0, 0.2, 1 μg/ml chloramphenicol or 0.02, 0.1, 0.3 μg/ml ciprofloxacin for *E*. *coli* co-cultures, or 0, 1, 3 μg/ml chloramphenicol for the *E*. *coli* and *S*. *typhimurium* co-culture. We imaged at least three positions per pad, resulting in measurements of hundreds of single cells for each position (for n values for each case see Fig. S2). 1.5% low melting agarose pads were made using M9 minimal medium containing 0.2% glycerol, 0.01% casamino acids, 0.15 μg/ml biotin, and 1.5 μM thiamine. Cells were diluted and mixed at ratios as indicated above and placed on pads containing 100 μM IPTG and chloramphenicol or ciprofloxacin. Images were taken using a Nikon Ti-E microscope with 100x objective lens for 130 mins at 5 min intervals. The temperature of the microscope chamber was held at 32 °C for the duration of the experiment.

### Data analysis

Images were analyzed in Matlab. We used the automated image processing package SuperSegger^30^ to measure cell growth rates and identify neighboring cells. An individual cell’s lineage starts just after its mother has divided, forming it and a sister cell, and it ends when the cell divides into two daughter cells. Growth rate is defined as the natural log of the ratio of the length of the cell at the end of the lineage to its length at the start of the lineage, divided by the length of the lineage in minutes. Thus, the growth rate is the exponential rate constant^31^. Custom Matlab scripts were used to analyze growth data and neighbor effects. Statistical analysis of growth rates was performed in Matlab.

### Toxicity experiments

To determine the antibiotic toxicity of the strains, we added a final concentration of 0, 0.1, 0.2, 0.5, 1, 2, 5, or 10 µg/ml of chloramphenicol or 0, 0.05, 0.1, 0.2, 0.5, 1, 2, or 5 µg/ml of ciprofloxacin to each culture. The samples were sealed with evaporation-limiting membranes (Thermo Scientific AB-0580) and grown in 96-well plates at 37 °C with orbital shaking at 200 rpm. OD600 readings were taken with a BioTek Synergy H1m plate reader every 10 m for 18 h. The toxicity curves represent change in growth for the first 2 h for consistency with the length of the microscopy experiments. All experiments were performed in triplicates with biological replicates.

### Mathematical model

To simulate cell growth with different neighbors in the presence of antibiotics, we used an agent-based model with Moore neighborhood architecture to describe the spatial interactions between cells and the environment (Fig. 4A)^32–34^. We represent each cell with two ordinary differential equations describing intracellular antibiotic concentration (Eq. 1) and cell biomass (Eq. 2). The model assumes exponential growth, which is valid for the short durations (~2 h) over which modeling and experiments are conducted. The biomass equation has a term for the toxicity of the environment, which is derived from Van Impe *et al*.^15,35,36^.

1 $$\begin{array}{ccc}\frac{d{C}_{in}}{dt} & = & \frac{1}{6}(\,{\sum }_{j=1}^{all\,adjacent\,Neighbors}(\frac{1}{2}{K}_{out,j}+\frac{1}{2}{K}_{in}){C}_{in,j}+{\sum }_{k=all\,adjacent\,Neighbors+1}^{4}{K}_{in}{C}_{out})\\  &  & +\,\frac{1}{12}(\,{\sum }_{j=1}^{all\,diagonal\,Neighbors}(\frac{1}{2}{K}_{out,j}+\frac{1}{2}{K}_{in})\times {C}_{in,j}+{\sum }_{k=all\,diagonal\,Neighbors+1}^{4}{K}_{in}{C}_{out})-{K}_{out}{C}_{in}\end{array}$$

2 $$\frac{dN}{dt}=\mu \cdot N\cdot (\frac{1}{1+{(\frac{{C}_{in}}{{K}_{c}})}^{{h}_{c}}})$$

The total antibiotic concentration at each time point is assumed to be equal to the antibiotic concentration in the environment and inside cells. We assume instantaneous diffusion within environments separated by a membrane.

3 $${C}_{total}={C}_{out}+\sum _{i=1}^{all\,cells}\,{C}_{in,i}$$

Our model focuses on the focal cell and its neighbors. C_in_ is the intracellular antibiotic concentration, and C_out_ is the extracellular concentration. N is biomass of the cell, and μ is the maximum growth rate. K_in_ and K_out_ are antibiotic entry and exit based on the presence of efflux pumps. We assume that if two cells are close together, the efflux from the neighbor will create a small area with a higher relative antibiotic concentration. We model this as the influx into the focal cell where an edge with a neighbor has an influx rate of $\,1/2\,{K}_{out,neighbor}+1/2\,{K}_{in}$. The first term represents the effect of the gradient produced by efflux from the neighboring cell with some loss to the environment and the second term represents passive influx that may occur. The second term sets a lower bound so that $\,1/2\,{K}_{out,neighbor}+1/2\,{K}_{in}\ge {K}_{in}$.

For the effect of antibiotics on change in biomass, we fit experimental data to a Hill function. Parameters for the toxicity term, h_c_ and K_c_, were fit to Δ*acrB* toxicity curves for chloramphenicol and ciprofloxacin (Fig. S1). For modeling cell growth under ciprofloxacin, we decreased K_out_ by using fits to experimental data. All model fits were conducted by minimizing least-squares error. All model parameters are listed in Table S2.

## Electronic supplementary material

Supplementary Information

Table S1

## Data Availability

The datasets generated during and/or analyzed during the current study are available from the corresponding author on reasonable request.
