# Supplementary material for: Antibiotic export by efflux pumps affects growth of neighboring bacteria
Source: Sci Rep. 2018 Oct 11;8:15120. doi: 10.1038/s41598-018-33275-4 (PMC6181935; doi:10.1038/s41598-018-33275-4)
Supplement: Supplementary file 2 — Table S1 [file 41598_2018_33275_MOESM2_ESM.docx]

**SUPPLEMENTARY INFORMATION**

**Antibiotic export by efflux pumps affects growth of neighboring bacteria**

Xi Wen, Ariel M. Langevin, Mary J. Dunlop

**
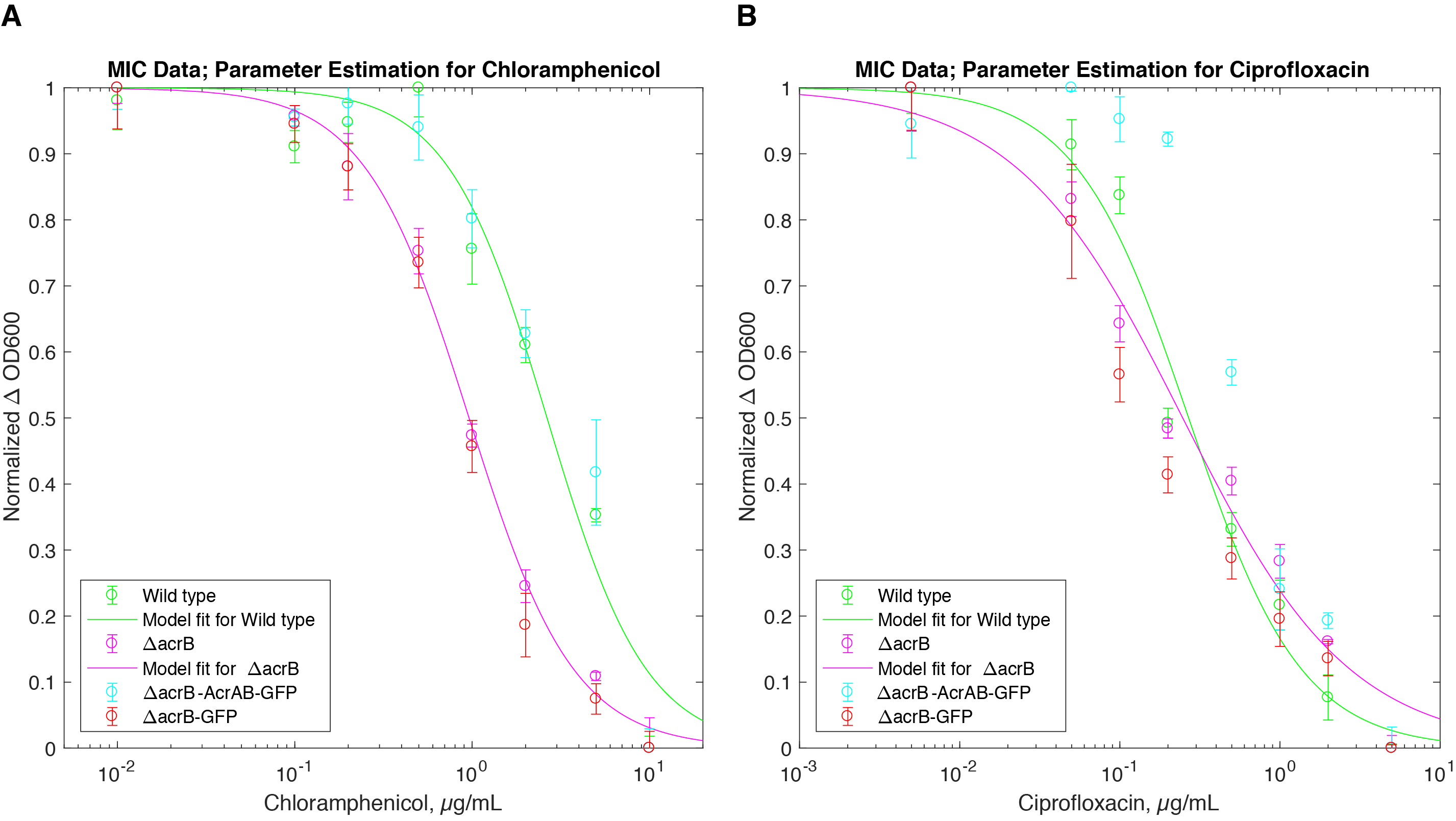
**

**Figure S1.** Toxicity curves and data fitting for model parameters. **(A)** Chloramphenicol and **(B)** ciprofloxacin experimental data for strains: wild type, Δ*acrB*, Δ*acrB*-AcrAB-GFP, and Δ*acrB*-GFP. Hill function fits for wild type and Δ*acrB* strains. Fits were conducted by minimizing least-squares error. Error bars show standard deviation of n = 3 biological replicates. Parameters for Δ*acrB* Hill function model fit are listed in Table S2.

**
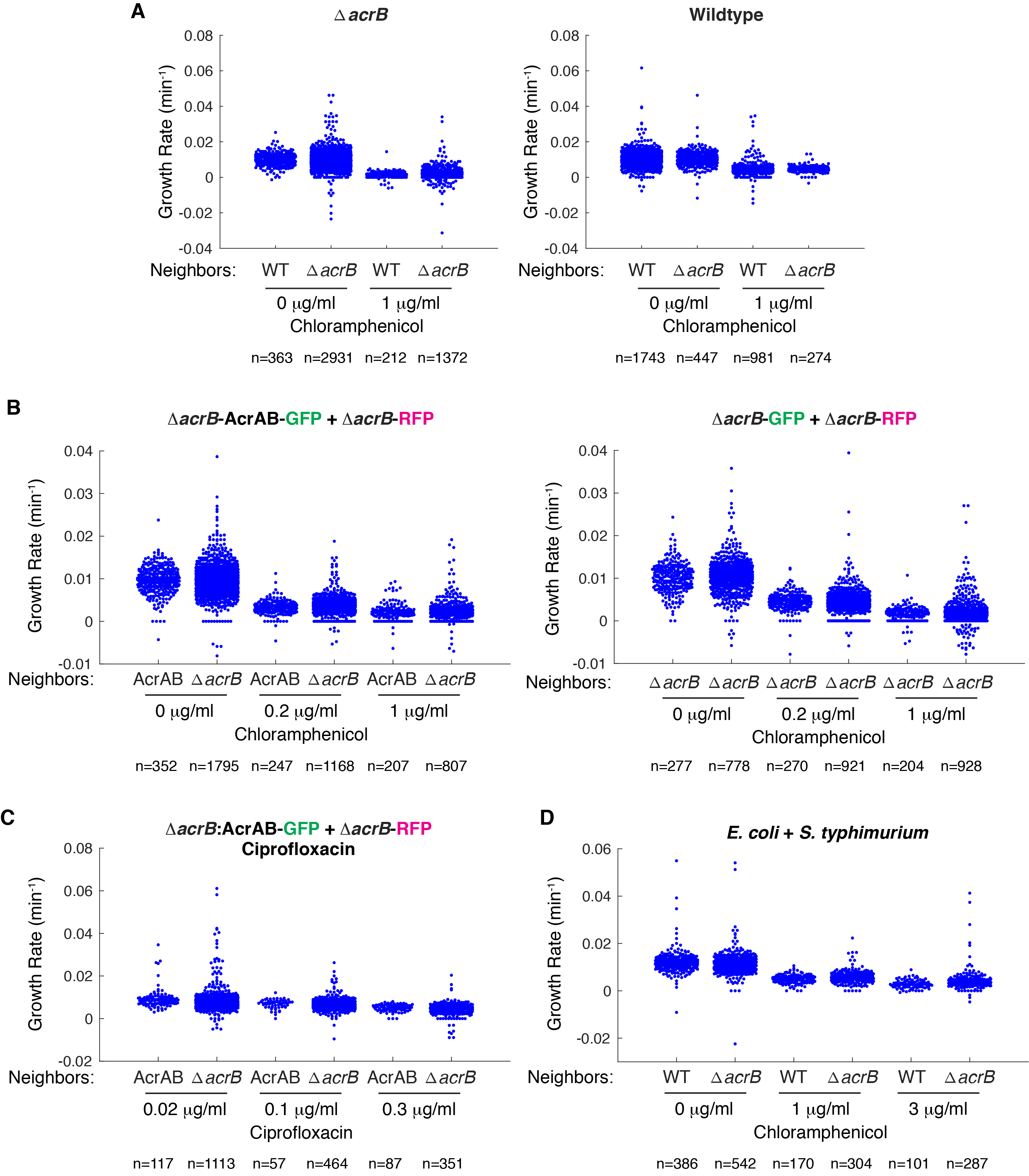
**

**Figure S2.** Full data sets for figures including outliers and number of cells (n). Data set corresponding to **(A)** Figs. 1B-C, **(B)** Figs. 2C-D, **(C)** Fig. 4E, and **(D)** Fig. 5. Each blue dot indicates the growth rate of a single cell. We note that in all cases, the plots shown in the main text include ≥97% of cells. The automated image analysis process occasionally calculates artificially high or low growth rates, but this is a rare occurrence (always <3%, but more typically <1% of cells). We include the full data sets here for completeness.

**
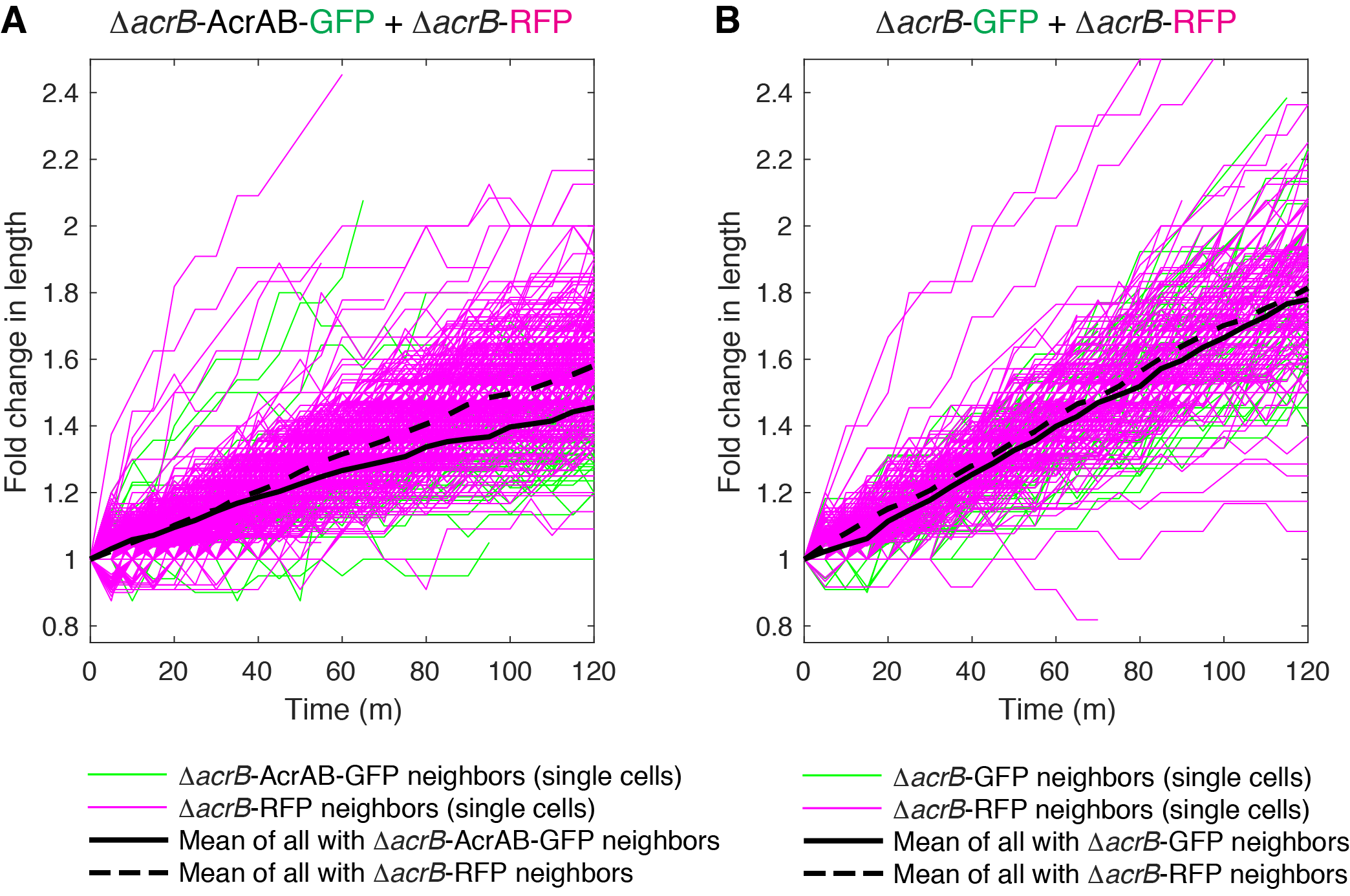
**

**Figure S3.** Fold change in cell length over time for all individual cells. **(A)** Δ*acrB-*RFP and AcrAB*-*GFP cells were mixed in ratios of 1:5 and 5:1 and grown on agarose pads with 0.2 µg/ml chloramphenicol. Colored lines show all cell traces and black lines show the mean values, as indicated in the figure legend. A fold change of two at the final time point indicates that a cell has doubled. **(B)** Δ*acrB-*RFP and Δ*acrB-*GFP cells for conditions as described in (A).

**
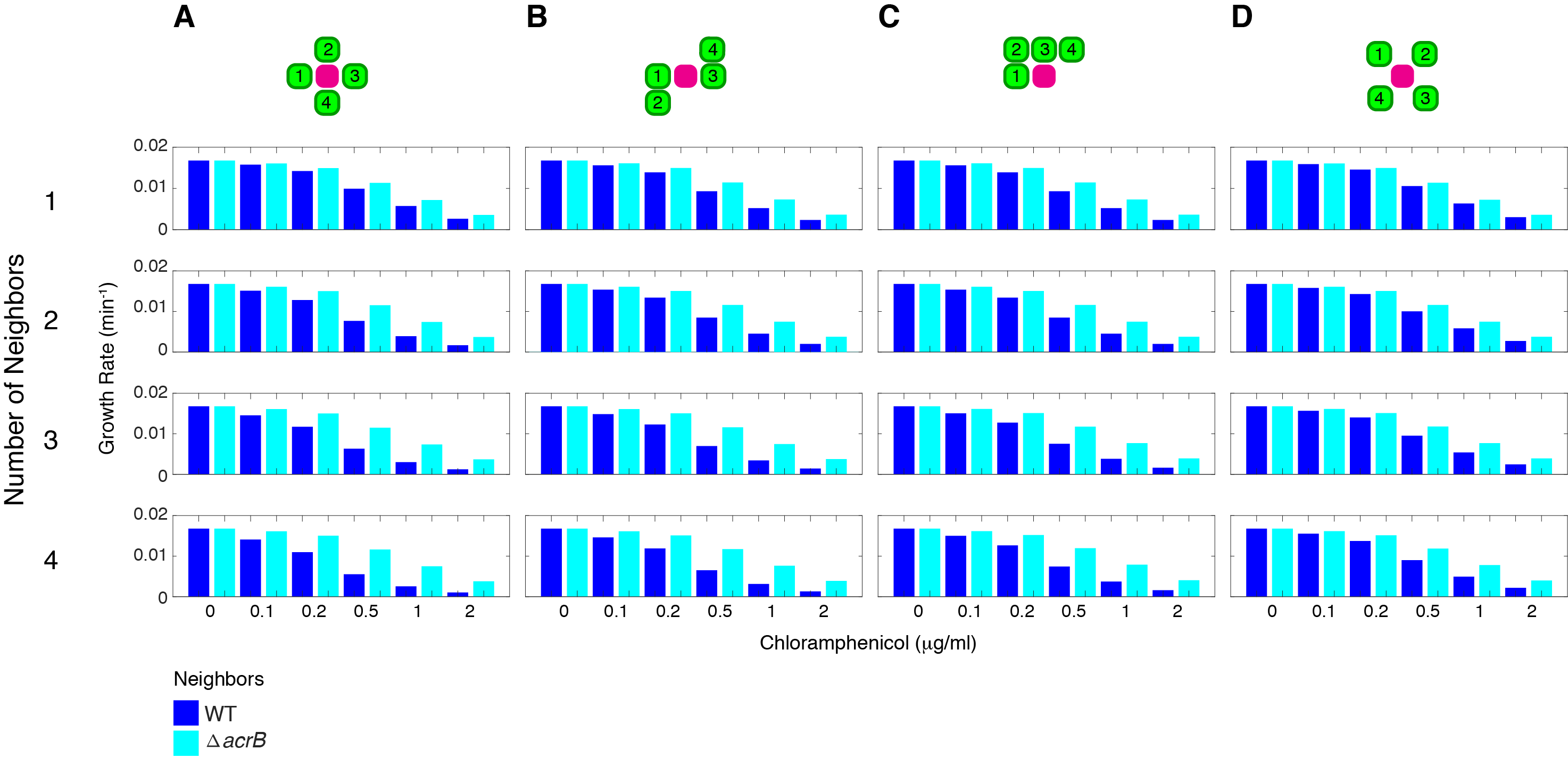
**

**Figure S4.** Impact of neighborhood on focal cell growth rate. **(A-D)** Each row represents a different number of neighbors and each column represents different neighborhood layouts. When the number of neighbors is one (first row) only the cell labeled “1” in the schematic is included in the simulation. For two neighbors, cells “1” and “2” are included, and so on. The neighborhoods evaluated are **(A)** spread out, but close to the focal cell, **(B)** neighbors are closer to each other, **(C)** neighbors are clustered around focal cell, and **(D)** spread out, but further from focal cell. The model was evaluated for Δ*acrB*-RFP cells with Δ*acrB*-AcrAB-GFP (blue) and Δ*acrB*-RFP (cyan) neighbors exposed to different concentrations of chloramphenicol.

**Table S1.** Mean, standard deviation, and p-values for growth rate data.

**Table S2.** Model parameters. Parameters for the model derived from data in Fig. S1, are calculated using from experimental doubling time, or are approximated based on the efflux efficiency (fold difference in the MIC) of different strains.

| **Parameter** | **Symbol** | **Units** | **Value** |
| --- | --- | --- | --- |
| Initial Cell Biomass | N(0) | rel. cell area | 1 |
| Initial Cell Antibiotic Concentration | C_in_(0) | µg/mL | 0 |
| **Chloramphenicol** | | | |
| Cell Doubling Time | T_d_ | min | 60 |
| Maximum Growth Rate | µ | rel. cell area min^-1^ | 0.1106 |
| Influx rate via Diffusion | K_in_ | min^-1^ | 1 |
| Efflux rate via Diffusion | K_out,Δ_*_acrB_* | min^-1^ | 1 |
| Efflux rate via Diffusion & Active Efflux | K_out,WT_ | min^-1^ | 4 |
| Repression coefficient | K_c_ | µg/mL | 0.960 |
| Hill coefficient | h_c_ | Dimensionless | 1.47 |
| **Ciprofloxacin** | | | |
| Cell Doubling Time | T_d_ | min | 90 |
| Maximum Growth Rate | µ | rel. cell area min^-1^ | 0.0737 |
| Influx rate via Diffusion | K_in_ | min^-1^ | 1 |
| Efflux rate via Diffusion | K_out,Δ_*_acrB_* | min^-1^ | 1 |
| Efflux rate via Diffusion & Active Efflux | K_out,WT_ | min^-1^ | 3 |
| Repression coefficient | K_c_ | µg/mL | 0.247 |
| Hill coefficient | h_c_ | Dimensionless | 0.83 |
